# Novel synergistic fungicidal mixtures of oxathiapiprolin protect sunflower seeds from downy mildew caused by *Plasmopara halstedii*

**DOI:** 10.1371/journal.pone.0222827

**Published:** 2019-09-23

**Authors:** Yigal Cohen, Avia E. Rubin, Mariana Galperin

**Affiliations:** The Mina & Everard Goodman Faculty of Life Sciences, Bar Ilan University, Ramat-Gan, Israel; Pennsylvania State University, UNITED STATES

## Abstract

Plenaris (oxathiapiprolin) applied to sunflower seedlings was highly effective in controlling downy mildew incited by the oomycete *Plasmopara halstedii*. In vitro assays revealed strong suppression of zoospore release and cystospore germination of *P*.*halstedii* by Plenaris. Bion (acibenzolar-S-methyl) and Apron (mefenoxam) were partially effective when used singly, but performed synergistically when mixed with Plenaris. Seed treatment (coating) with Plenaris provided dose-dependent control of the disease whereas Bion and Apron provided partial or poor control. However, seeds treated with mixtures containing reduced rates of Plenaris and full rates of Bion and/or Apron provided complete control of the disease due to the synergistic interaction between these components. Such mixtures should be used for seed treatment in the field to minimize selection pressure imposed on the pathogen.

## Introduction

Sunflower downy mildew (SDM) incited by the biotrophic oomycete *Plasmopara halstedii* (Farl.) Berl. and de Toni is a devastating disease of sunflower (*Helianthus annuus* L) worldwide [1–3]. SDM has been reported from over 50 countries [4] except Australia and New Zealand. First epidemics in cultivated sunflower were reported in the US in 1890, nearly 50 years before the first records of the pathogen were reported in Europe [3]. The long distance dispersal of SDM almost certainly occurred through the exchange of oospore-contaminated seeds [5, 6].

This oomycete is homothallic [7]. Young sunflower seedlings inoculated with sporangia of *P*. *halstedii* produce abundant sporangia on their cotyledons within one week and many oospores within about 10 days (whose size is 43.4 ±5.2 μm) are produced in their hypocotyl and roots (Y. Cohen, unpublished). Zoospores, released from sporangia produced by germinating soil-borne oospores, attack the roots of the emerging sunflower plants in the spring [8] and cause latent or systemic infection with downy mildew. Symptomatic plants produce abundant asexual sporangia that infect leaves and apical meristems of other plants, causing systemic symptoms [5, 9, 10]. Symptomatic and non-symptomatic plants might produce oospores in their root, shoot and seeds. These oospores incorporate into the soil and may cause infection of the new crops in subsequent years [5–7, 10].

Oospores are the indispensable key element for overwintering in the soil and for long distance dispersal via seed contamination [5–7]. In particular, the uncontrolled exchange of oospore-contaminated seeds is a major reason for the nearly worldwide distribution of the pathogen since the middle of the 20^th^ century [3].

The genome of *P*. *halstedii* has been recently sequenced and more than 600 putative effector genes were identified [11] meaning that this pathogen invests about 4% of its total protein in pathogenicity-related peptides. Effector polymorphisms were used to identify pathotypes of *P*. *halstedii* from the field [12]. Within the secretome of *P*. *halstedii*, the majority of putative effector genes belong to the families of RxLR (Arg-Xaa-Leu-Arg) and CRN (*Crinkler* gene family) candidates [11]. These authors also identified a broad spectrum of proteins with putatively pathogenicity related function such as cutinases, pectinases, and phospholipases.

The high genetic variability of the pathogen [13, 14], its ability to recombine parasexually [15], and the introduction of new host-resistance genes, enhanced the evolution of new, virulent races of the pathogen. In a recent review, Viranyi et al. [1] reported on the occurrence of 24 races of *P*. *halstedii* in Europe (including Turkey) and 41 races in America. They recognized a gradual shift from the early low virulent pathotypes such as 100, 300 or 700 towards pathotypes with higher virulence such as 310, 330, 710 or 730. Iwebor et al. [16] recently reported on three new races in Russia, also new to Asia, that have appeared after 2016.

The rapid evolution of new races that overcame host-resistance genes led seed companies to use phenylamide fungicides such as Apron (metalaxyl, mefenoxam = MFX) for seed coating to prevent primary infection. However, like many other oomycete pathogens [17], *P*. *halstedii* quickly developed tolerant genotypes against phenylamides in France [18], Spain [19], and Germany [20]. The widespread resistance to phenylamides [21] called for new fungicides with new mode of action to be used.

A novel anti oomycete fungicide that can successfully protect sunflower against downy mildew is oxathiapiprolin. Oxathiapiprolin (OXPT) is a new piperidinyl thiazole isoxazoline fungicide (FRAC code U15) with extremely high activity against a range of plant pathogenic oomycetes except *Pythium* (see literature cited by Cohen [22]). Oxathiapiprolin acts at multiple stages of the pathogen's asexual life cycle at extremely low concentrations (99% inhibition of zoospore release and sporulation at 0.00001 and 0.01 ppm ai, respectively [22]). Preventatively, it inhibits zoospore release zoospore motility, cystospore germination and direct germination of sporangia. Curatively, it stops mycelial growth within the host plant before visible lesions occur, offering protection at one and two days post-infection. It stops mycelial growth, inhibits further lesion expansion, and inhibits spore production. It shows translaminar and acropetally systemic movement, protecting treated leaves and newly developing leaves as they emerge and grow [22].

The molecular target of OXPT is the oxysterol binding protein OSBP [23], a member of the OSBP-related protein family of lipid transfer proteins. They constitute a family of sterol and phosphoinositide binding and transfer proteins in eukaryotes. The lipid-binding proteins are implicated in many cellular processes related to oxysterol, including signaling, vesicular trafficking, lipid metabolism, and non-vesicular sterol transport. Oxysterol-binding protein localizes to endoplasmic reticulum-Golgi contact sites, where it transports cholesterol and phosphatidylinositol-4-phosphate and activates lipid transport and biosynthetic activities [24].

The data presented by Pasteris et al. [23] indicate that OXPT is a high-risk fungicide that requires careful use in the field to prevent the development of resistant mutant isolates. Resistance against OXPT was induced in *Phytophthora capsici* by UV irradiation [25]. Miao et al. [26] re-confirmed that three point mutations confer resistance to OXPT in *P*. *capsici*. They also found a new point mutation which produces OXPT resistance in both *P*. *capsici* and *P*. *sojae*. Mutants with any of these three point mutations may have survival potential in the field [26].

According to FRAC (http://www.frac.info/), the resistance risk of OXTP is medium to high and therefore resistance management is required. Such management can include mixing the fungicide at risk with another fungicide with a different mode of action [17].

Oxathiapiprolin mixtures (with azoxystrobin, mandipropamid or mefenoxam) were reported to be highly effective against *Phytophthora infestans* in tomato [27] and *Pseudoperonospora cubensis* in cucumber [28]. Interestingly, the mixture of OXPT+mefenoxam was also effective against a mefenoxam-resistant isolates of these pathogens.

In the present research, two fungicides were selected as partners for mixing with oxathiapiprolin: Bion and Mefenoxam. Bion, [acibenzolar-S-methyl, (ASM) or BTH, is a chemical inducer that activates systemic acquired resistance (SAR) in plants. It acts at the site downstream of salicylic acid accumulation and induce the PR-1a promoter [29]. It protects sunflower against downy mildew [30–32] and proved effective in the control of downy mildew in tobacco [33] and powdery mildew in cucumber [34]. Apron (mefenoxam, MFX) applied as seed treatment was effective against sunflower downy mildew until resistant isolates of *P*. *halstedii* dominated the fungal population [21].

The major objective of this study was to examine the efficacy of seed treatment with Oxathiapiprolin, Bion and Mefenoxam against SDM with the aim of identifying synergistic mixtures that will reduce the selection pressure imposed on the pathogen by oxathiapiprolin. For this purpose, we used different cultivars of sunflower, different isolates of *P*. *halstedii* and different inoculation methods.

## Materials and methods

### Plants

Three cultivars of sunflower (*Helianthus annus* L) were used: (1) Bacardi F1 (obtained from Dr. Jennifer Foster, Syngenta Seed Care, Basel, Switzerland), oil type, seed weight 60 mg per seed; (2) June (bought in the market place, Ashdod, Israel), confection type, 80 mg per seed; (3) Hazera 882 (obtained from Dr. Yossi Buskila, Hazera Seeds, Mivhor, Israel), confection type, 160 mg per seed.

### Pathogen

Thirteen isolates of *Plasmopara halstedii* were used. Their origin and properties are shown in Table 1. The pathogen was propagated on sunflower seedlings once every 7–10 days using the whole seedling inoculation (WSI) method (see below). Fresh sporangia were collected from inoculated seedlings at 7–10 dpi (days post inoculation) and used for inoculations.

**Table 1:** Isolates of *Plasmopara halstedii* used in this study.

Isolate	Race	Year collected	Region	Country
**1978R**	710	2014	Andalusia	Spain
**DBO**	700	2014	Andalusia	Spain
**1SM**	304	2014	Andalusia	Spain
**Ph1-15**	310	2014	Andalusia	Spain
**330**	330	2015	Krasnodar	Russia
**334**	334	2016	Krasnodar	Russia
**710**	710	2015	Krasnodar	Russia
**730**	730	2016	Krasnodar	Russia
**T4**	?	2017	Edirne	Turkey
**Basel**	?	?	?	Switzerland

### Inoculation

Three methods of inoculation were employed, depending on the experiment:

#### (i) Whole seedling inoculation (WSI)

Cohen and Sackston [5] first described this method. Briefly, sunflower seeds were washed with running tap water for 30 min and placed on a wet filter paper in closed plates for 3 days at 20°C in the dark for germination. The germinated seedlings were then inoculated by immersion in sporangial suspension (one seedling per ml, about 50,000 sporangia per ml) of *P*. *halstedii* for 6 h at 18°C. The inoculated seedlings were then planted in 250 ml pots filled with pasteurized soil mixture (peat: perlite, 10:1, v/v), 8–10 seedlings per pot. The potted plants were incubated in a growth chamber at 23°C under continuous cool white fluorescent light (100 μmole.m^2^.s^-1^) for 6 days to allow for fungal colonization and thereafter, in a dew chamber at 18°C in the dark to allow for fungal sporulation. Seven hours of incubation in a dew chamber (18°C, darkness) were required for sporulation of *P*. *halstedii* on such infected plants. The proportion of seedlings with fungal sporulation on their cotyledon leaves was determined at 7 dpi.

#### (ii) Spray inoculation

Potted sunflower plants at the cotyledon stage (7–8 days after sowing) were sprayed on the upper leaf surface with sporangial suspension (as above), placed in a dew chamber (18°C, darkness) for 15 h to ensure infection and, thereafter at 23°C under continuous illumination for 6 days (as above) to facilitate colonization. At the end of the colonization period, the plants were transferred to a dew chamber (18°C, darkness, 7 h) to induce sporulation of the pathogen.

#### (iii) Soil inoculation

Sunflower seeds were planted in 250 ml pots containing pasteurized soil mixture, 10 seeds per pot and incubated at 23°C. At 3 days after sowing, 10 ml of sporangial suspension (about 50,000 sporangia/ml) were poured onto the soil surface of each pot as previously described [5]. Pots were incubated at 18°C in the dark for 15 h to ensure infection and, thereafter at 23°C under continuous illumination for 6 days to facilitate colonization. At the end of the colonization period, the plants were transferred to a dew chamber (18°C, darkness, 7 h) to induce sporulation of the pathogen.

### Fungicides

The following three fungicides were used, all obtained from Syngenta Seed Care, Basel, Switzerland: Plenaris 20 SC, containing 20% oxathiapiprolin, Bion 35 SC, containing 35% Acibenzolar-S-Methyl [benzo (1, 2, and 3) thiadiazole-7-carbo-thioic acid S-methyl ester = BTH], and Apron 37.5 SL, containing 37.5% mefenoxam. Fungicides were suspended in water, diluted in water to various concentrations (always given as ppm active ingredient, ai) and used for WSI, foliar spray or soil drench.

### Seed treatment

The appropriate weight of a fungicide (s) was pipetted into a one-liter round-bottom glass flask, 1 ml of water was added and 1000 seeds were inserted into the flask. The flask was mechanically rotated for 10 min at a speed 100 rpm. The seeds were taken out, placed on a paper towel on the bench to dry for one day and thereafter kept in paper bags at 4°C until use.

Seeds were treated with fungicides with the premise that the quantity of Plenaris, Bion and Apron applied per seed should not exceed 30, 31 and 66 μg ai per seed, respectively (Syngenta, personal communication). Seeds were treated with gradually decreased quantities of Plenaris (18.8–0.78 μg ai /seed) and a single dose of Bion (25 or 31 μg ai /seed), Apron (50 or 66 μg ai /seed), or both. For comparison purposes, seeds were treated with Bion solo, Apron solo, or a mixture of both. Mixtures of Plenaris+Bion, Plenaris+Apron or Plenaris+Bion+Apron of various ratios (see below) were tested at doses of 0.0001–10 ppm ai for efficacy against infection of various isolates of the pathogen using the WSI method. Two cultivars were used; Bacardi (oil type 60 mg/seed) and June (confection type, 80 mg/seed).

### Data analysis

Experiments were repeated twice or more. Four pots with 8–10 plants per pot, per fungicide dose, were used for each isolate in each experiment. In spray and WSI experiments, percent-infected plants were transformed to percent inhibition of the disease and subjected to probit analysis using SPSS software. ED90-observed values (ED90obs) were deduced and used to calculate the expected ED90 values (ED90exp) by using the Wadley formula.[35, 36] Synergy factor (SF) was calculated as SF = ED90exp/ED90obs. In seed treatment experiments the expected efficacy was calculated by using the Abbott formula and the synergy factor was calculated as SF = observed efficacy/expected efficacy. In both methods, SF values greater than one indicated a synergistic interaction between the fungicides composing a mixture.

## Results

### Effect of fungicides on zoospore release, zoospore motility, and cystospore germination

**Fig 1:**
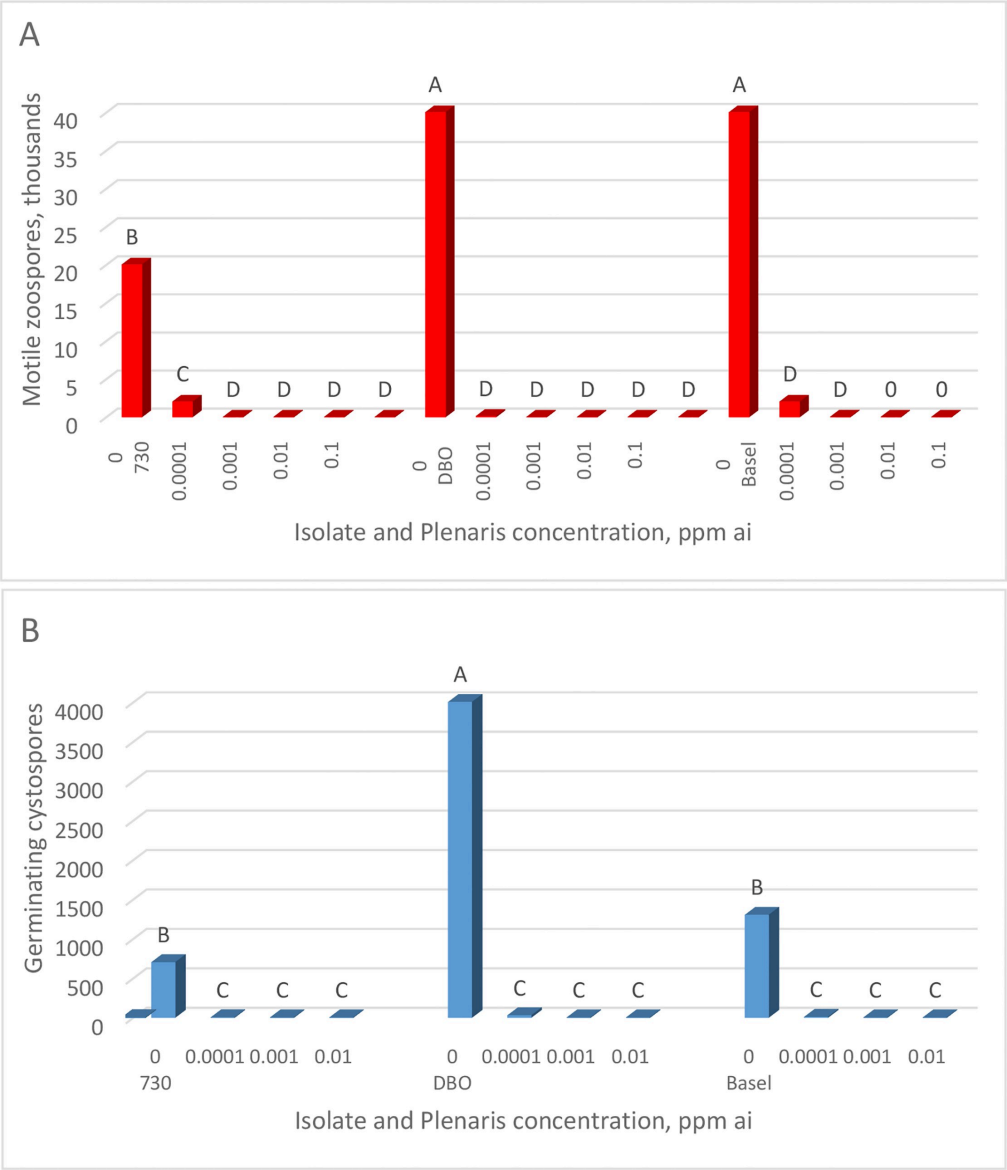
The effect of Plenaris on: A- Zoospore release. B- Cystospore germination. Sporangia of *Plasmopara halstedii* were incubated at 15°C in water containing various concentrations of Plenaris and examined microscopically after 2 h for zoospore release and after 20h for cystospore germination. Different letters on bars indicate on significant differences between treatments at α = 0.05.

**Fig 2:**
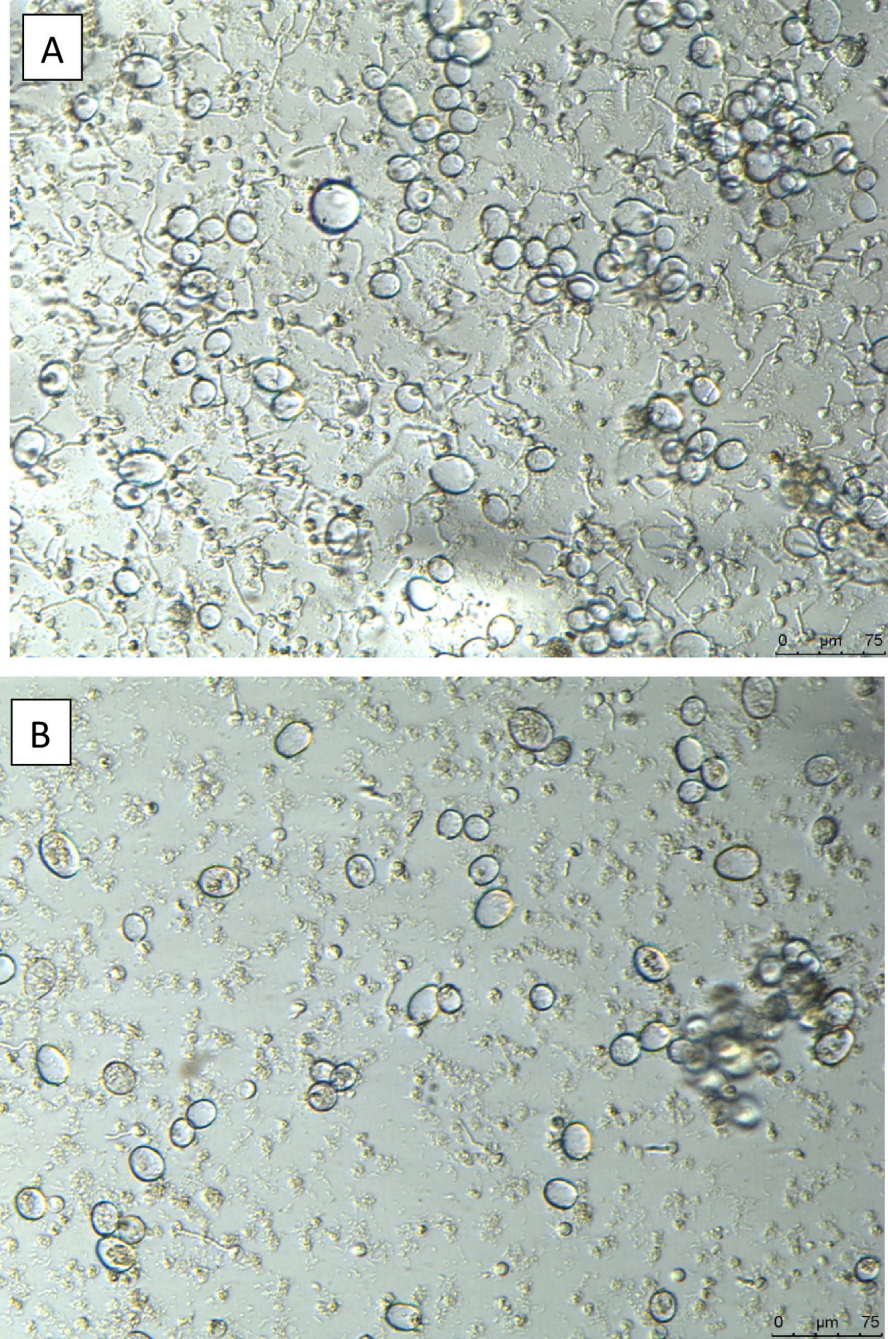
Cystospore germination of *Plasmopara halstedii* in: A- water. B- in Plenaries of 0.0001 ppm ai. Note the empty sporangia and germinating cystospores in A and empty sporangia and disintegrated cystospores in B.

Plenaris applied to a sporangial suspension of *P*. *halstedii* at 15°C strongly suppressed zoospore release from sporangia. Complete inhibition occurred at 0.001 ppm ai (Fig 1A). Motile zoospores that were subjected to Plenaries of 0.0001–10 ppm ai continue to swim for an hour, suggesting no effect of Plenaris on zoospore motility. The effect of Plenaris on cystospore germination is shown in Fig 1B. Complete inhibition of germination occurred at Plenaris of 0.0001 ppm ai. Micrographs showing the effect of Plenaris on cystospore germination are shown in Fig 2. Whereas most cystospores germinated in water (Fig 2A), almost no cystospore germinated in Plenaris of 0.0001 ppm ai (Fig 2B). Bion and Apron had no effect on zoospore release, zoospore motility, or cystospore germination at concentrations of 0.001–10 ppm ai.

### Effect of fungicides on infection: WSI method

**Fig 3:**
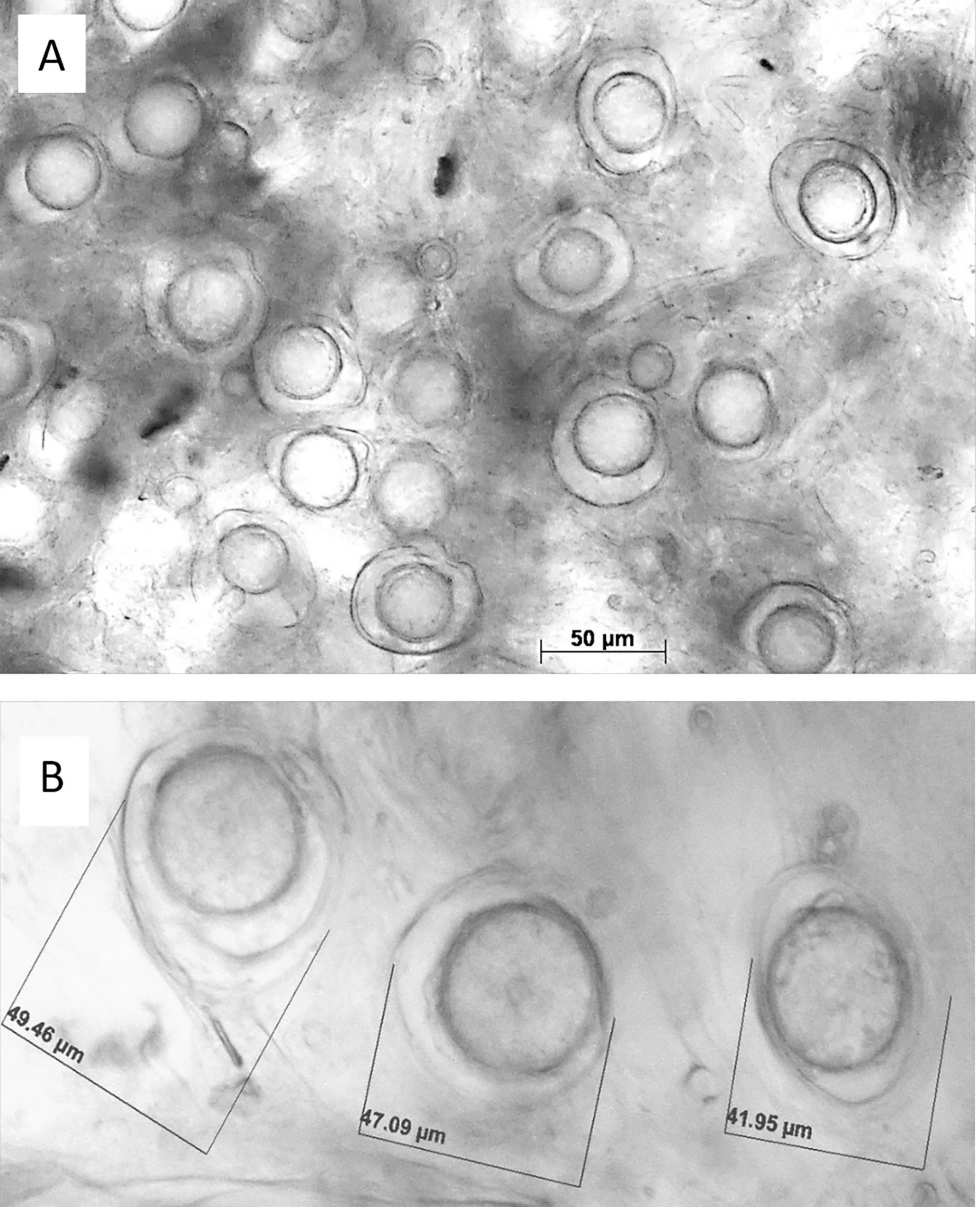
Oospores of *Plasmopara halstedii* produced in root and hypocotyl tissues of sunflower seedlings insulated by the WSI (whole seedling inoculation) method.

The successful infection of 3-days old untreated sunflower seedlings occurred within 3h of immersion in sporangial suspension (WSI method, Cohen and Sackston [9]). At 7 dpi, such infected seedlings showed abundant sporulation of *P*. *halstedii* on their cotyledon leaves after incubation in a dew chamber at 18°C for 7 h. At 12–14 dpi, many sexual spores, oospores, 43.4±5.2 μm in diameter were developed in the root and the bottom hypocotyl tissues of such infected plants (Fig 3).

**Fig 4:**
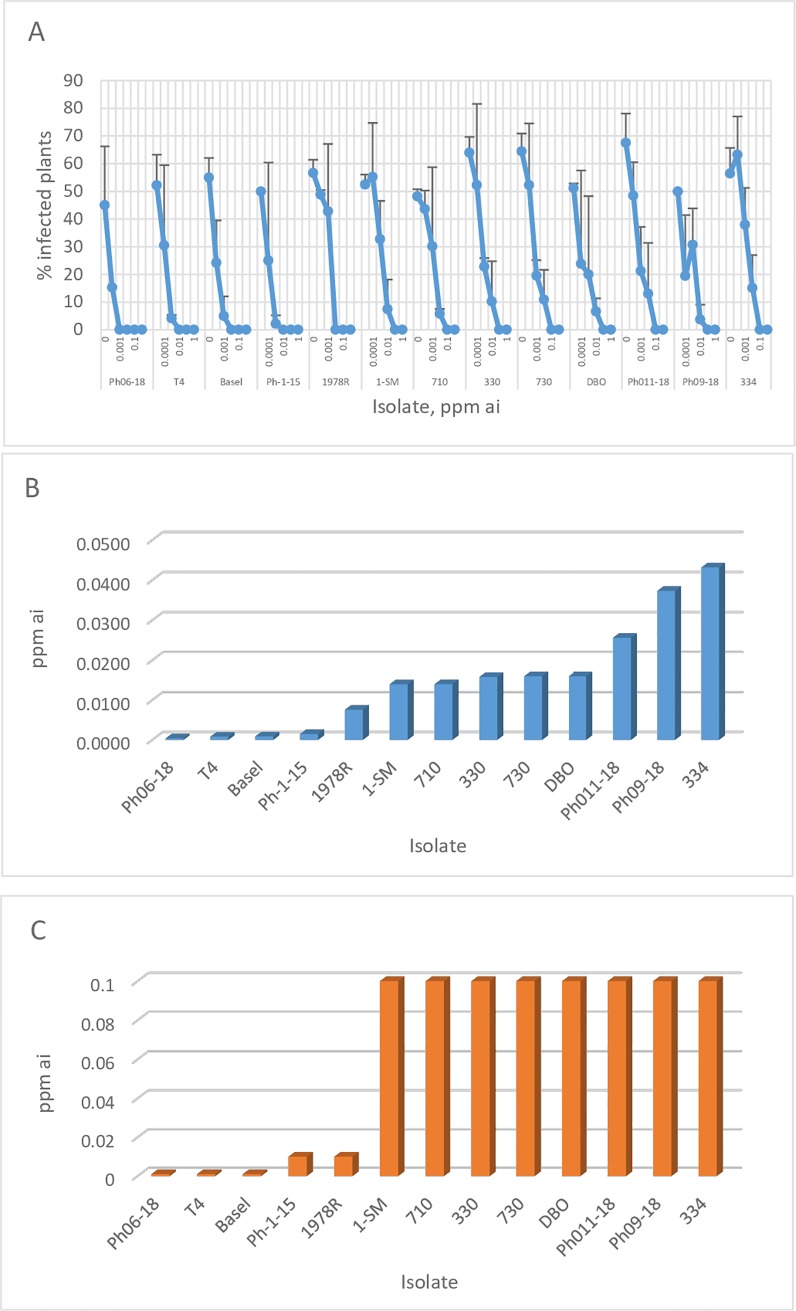
Dose-dependent efficacy of Plenaris in controlling each of 13 isolates of *Plasmopara halstedii* with the WSI method. Bars represent standard deviation of the mean. A- Percent infected plants. B- ED90 values (ppm ai) derived from probit analysis. C- MIC values (ppm ai) derived from values shown in A.

The efficacy of Plenaris applied at 0.0001–1 ppm ai (×10 fold dilutions) in controlling 13 isolates of *P*. *halstedii* inoculated by the WSI method is shown in Fig 4A. The ED90 values (dose required to suppress disease development by 90% relative to untreated inoculated plants) calculated by probit analysis ranged between 0.00039 and 0.04318 ppm ai (Fig 4B). The minimal inhibitory concentration (MIC) values of three, two, and eight isolates were 0.001, 0.01 and 0.1 ppm ai, respectively (Fig 4C).

Much higher doses of Bion or Apron were required to control the disease in sunflower seedlings inoculated with the WSI method. Thus, MIC values of 1, 10, 100 and >100 ppm ai of Bion were obtained for one, 2, 4 and 6 isolates, respectively, and MIC values of 10, 100 and >100 ppm ai of Apron were recorded for 4, 8 and 1 isolates, respectively. No association was noticed between the sensitivity levels of the isolates to the three fungicides.

**Fig 5:**
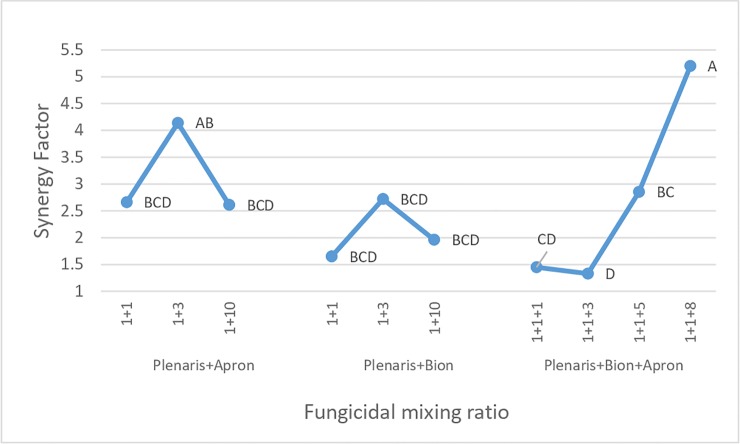
Synergy between Plenaris, Bion and Apron in controlling downy mildew in sunflower seedlings (WSI method, mean of 4–5 experiments per mixture). Synergy factor was calculated according to the Wadley formula.[35, 36] Different letters on curves indicate on significant differences between treatments at α = 0.05.

Results in Fig 5 show that Plenaris interacted synergistically (SF>1) with Apron, Bion and both, providing better disease control than expected, suggesting that a low dose of Plenaris in a mixture may provide as effective disease control as a full dose applied solo. No synergy occurred when the proportion of Plenaris in a mixture was lower than 5% (ratio of 1+20 or 1+1+20).

### Effect of fungicides on infection: Foliar spray

Seven-day-old plants at their cotyledon stage were sprayed with various doses of Plenaris (0.001–1 ppm ai), Bion (0.1–100 ppm ai) or Apron (1–1000 ppm ai), and thereafter inoculated with sporangia of either one of 13 isolates of *P*. *halstedii*. Results showed high control efficacy of Plenaris (mean ED90 = 0.003 ppm ai), low control efficacy of Bion (mean ED90 = 146.6 ppm ai) and quite poor control efficacy of Apron (mean ED90 = 522.8 ppm ai). Plenaris applied at 1dpi exhibited curative efficacy, with an ED90 value of about 10 ppm ai.

**Fig 6:**
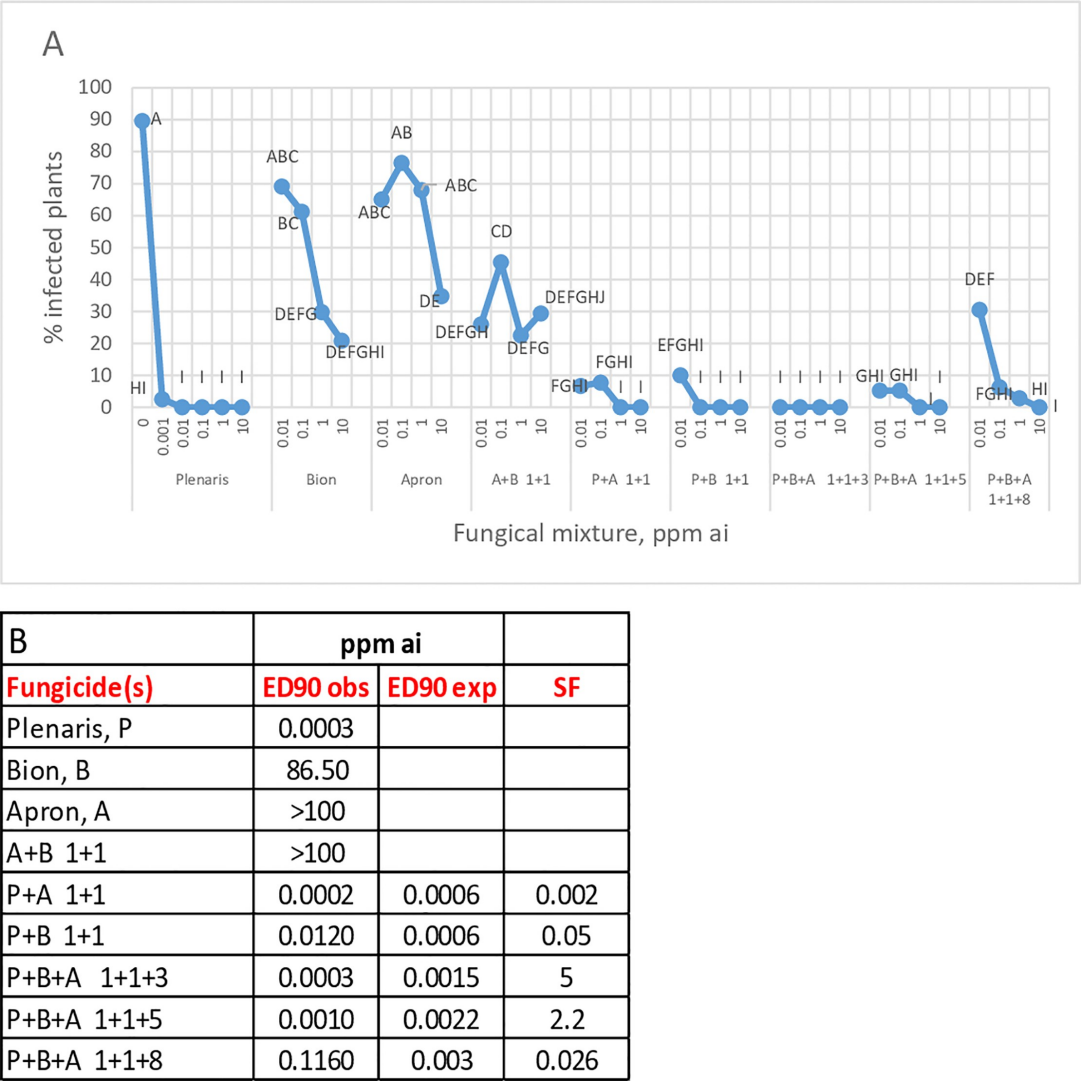
Efficacy of fungicidal foliar sprays in controlling downy mildew in sunflower seedlings. Mean of two experiments with Isolate 1-SM. A- Percent infected plant. Different letters on bars indicate on significant differences between treatments at α = 0.05. B- Synergy factors (calculated by the Wadley method) of fungicidal mixtures.

Mixtures of various ratios of the three fungicides were sprayed on 7-day old plants at their cotyledon stage and thereafter inoculated with isolate 1-SM of the pathogen. The results (mean of two experiments) are shown in Fig 6A. A mixture of Plenaris+Bion+Apron at a ratio of 1+1+3 and 1+1+5 (ai) performed better than expected, exhibiting SF values of 5 and 2.2, respectively (Fig 6B)

### Efficacy of seed treatment- Bacardi seeds

**Fig 7:**
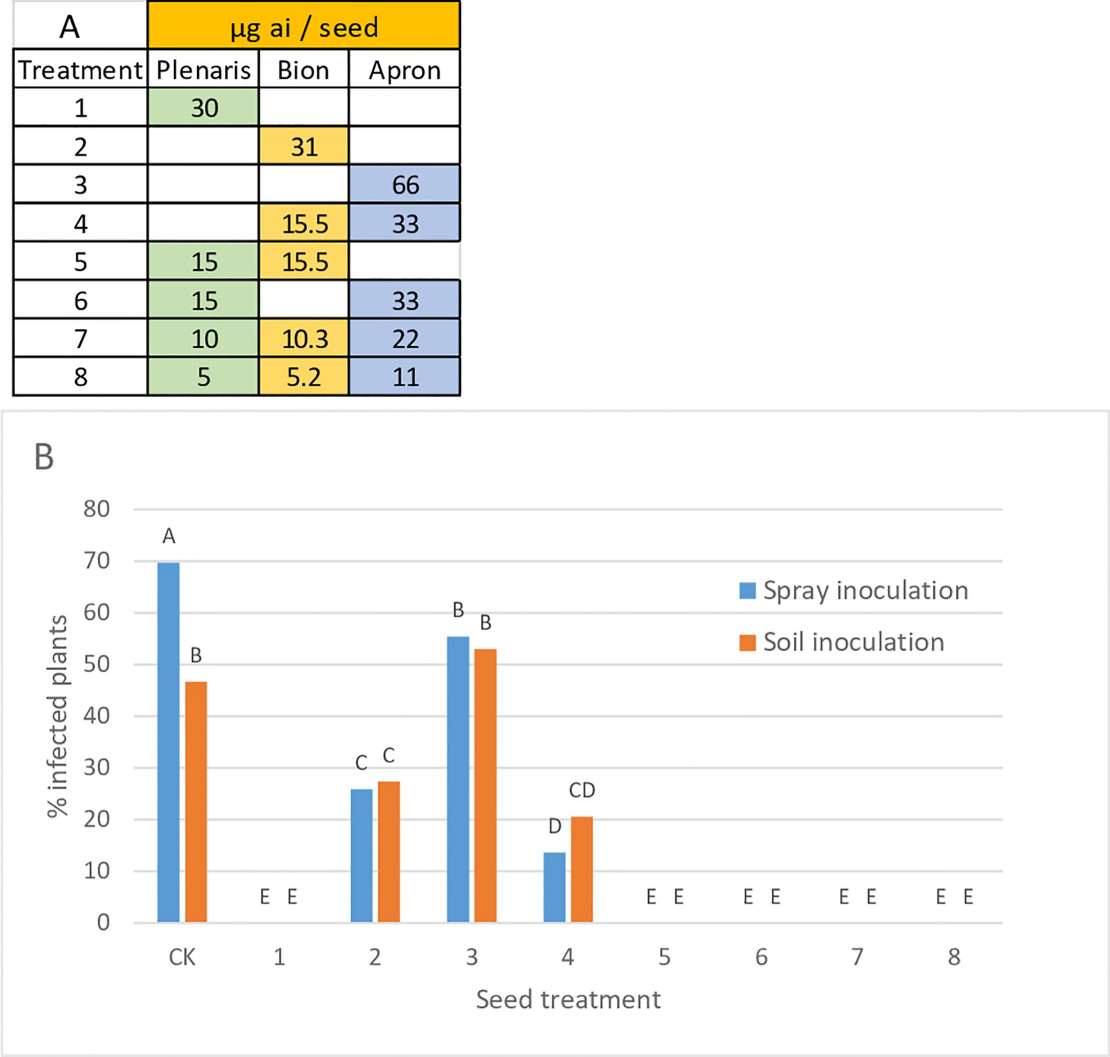
Control of downy mildew in sunflower cultivar Bacardi by seed treatment with Plenaris, Bion and Apron. Mean of three experiment. A- Doses of the fungicides applied per seed. B- Percent plants showing sporulation of *Plasmopara halstedii* at 7 dpi. Different letters on bars indicate on significant differences between treatments at α = 0.05.

Bacardi seeds (oil type, about 60 mg/seed) were treated with various combinations of Plenaris, Bion and Apron (Fig 7A), and inoculated with isolate 1-SM by either pouring sporangial suspension to the soil surface at 3 days after sowing (pre-emergence inoculation), or by spray inoculation of the developed cotyledon leaves at 7 days after sowing. Results in Fig 7B (mean of three experiments) show that both methods of inoculation were similarly effective. Plenaris at a dose of 30 μg ai/seed provided complete control of the disease (zero seedlings showing sporulation) while Bion at 31 μg ai /seed, Apron at 66 μg ai/seed, and Bion+Apron at half dose each (treatments 2–4), provided partial, though significant, control of the disease. Two-way and three-way mixtures containing reduced rates of the fungicides (treatments 5–8), provided complete suppression of disease development (Fig 7B).

**Fig 8:**
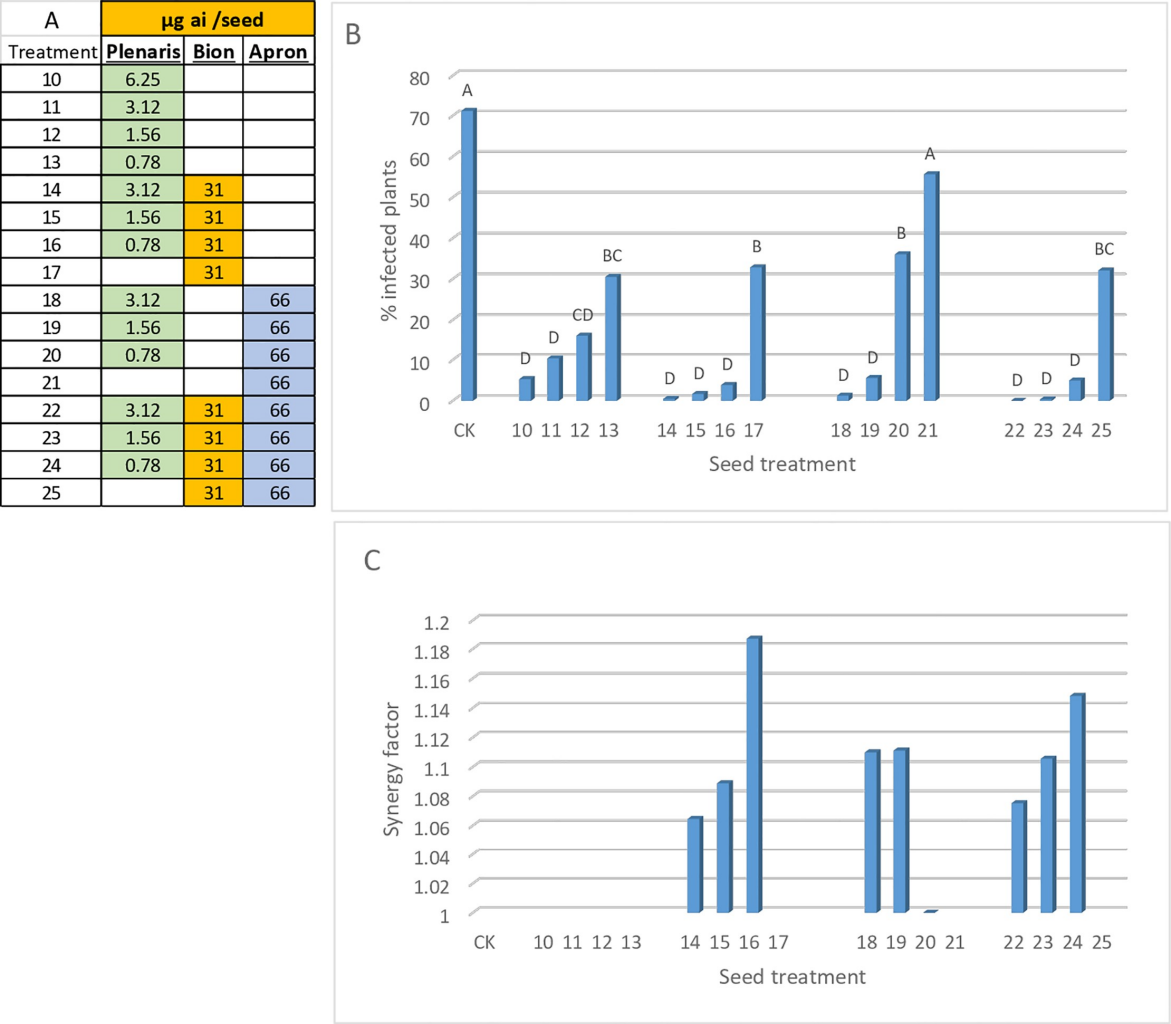
Control of downy mildew in sunflower cultivar Bacardi by seed treatment with Plenaris, Bion and Apron. Mean of 11 experiments. A- Doses of the fungicides applied per seed. B- Percent plants showing sporulation of *Plasmopara halstedii* at 7 dpi. Different letters on bars indicate on a significant differences between treatments at α = 0.05. C- Synergy factor calculated for the mixtures according to the Abbott formula [35, 36].

Results in Fig 8A and 8B (mean of 11 experiments) show the decreasing protective capacity of Plenaris at decreasing doses from 6.25 to 0.78 μg ai/seed (treatments 10–13). Bion, Apron and Bion+Apron at full dose (treatments 17, 21, and 25, respectively) were poorly effective (Fig 8B). Combining reduced doses of Plenaris with a full dose of Bion (treatments 14–16), Apron (treatments 18–20), or both (treatments 22–24) enhanced control of the disease. At its lowest dose of 0.78 μg ai/seed, Plenaris interacted better with Bion (treatment 16) than with Apron (treatment 20) (Fig 8B). The Abbott formula [35, 36] enabled calculation of the synergistic interaction between the three fungicides (Fig 8C). All mixtures with Bion (treatments 14–16), Apron (treatments 18 and 19, but not 20), or both (treatments 22–24), were synergistic (SF>1). SF usually increased as the dose of Plenaris decreased.

### Efficacy of seed treatments- June seeds

**Fig 9:**
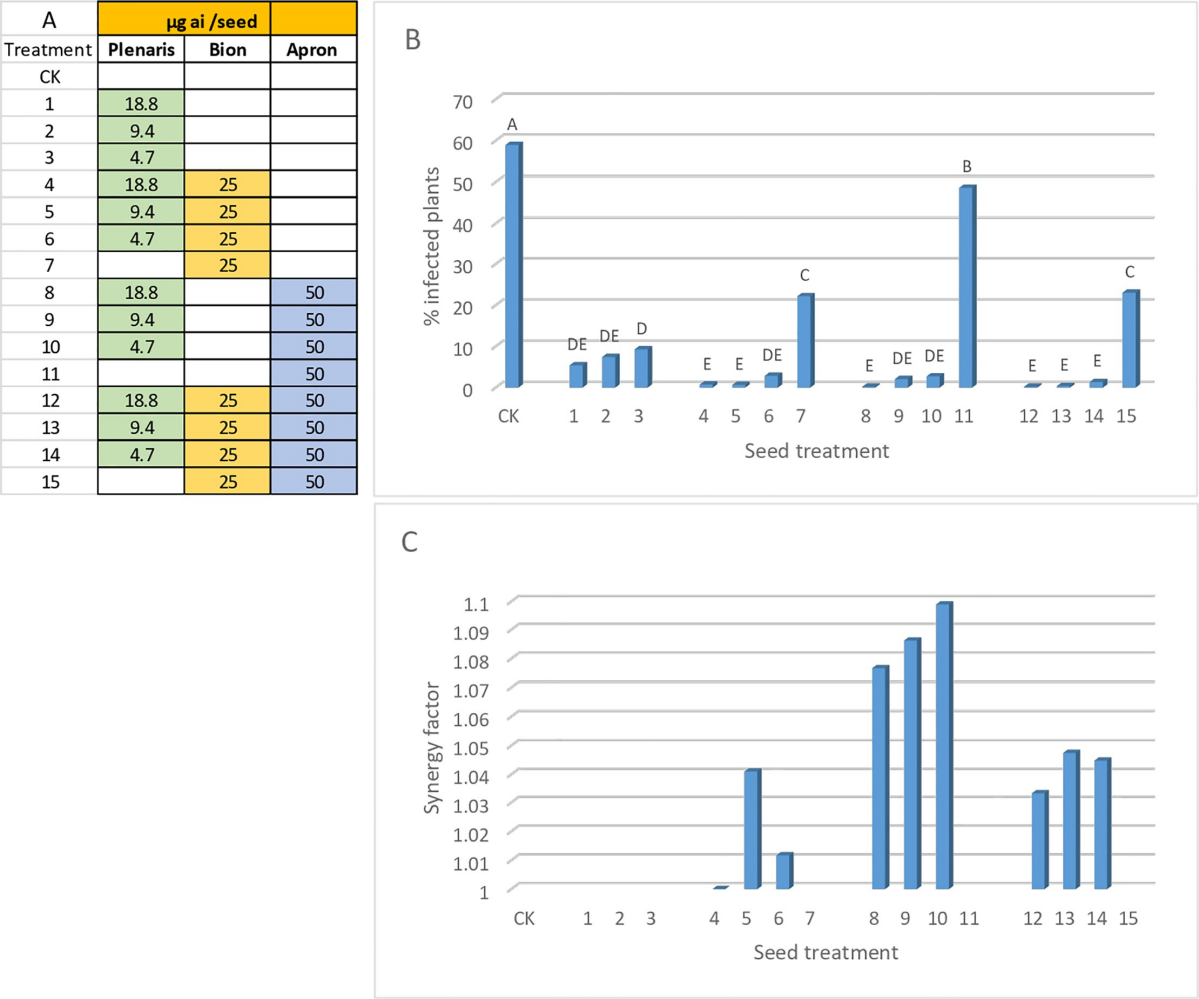
Control of downy mildew in sunflower cultivar June by seed treatment with Plenaris, Bion and Apron. Mean of 13 experiments. A- Doses of the fungicides applied per seed. B- Percent plants showing sporulation of *Plasmopara halstedii* at 7 dpi. Different letters on bars indicate on a significant difference between treatments at α = 0.05. C- Synergy factor calculated for the mixtures according to Abbott formula.

June seeds (confection type, about 80 mg/seed) were treated with various combinations of Plenaris, Bion and/or Apron (Fig 9A). At 7 days after sowing plants were spray inoculated with various isolates of *P*. *halstedii*. Results in Fig 9B (mean of 13 experiments) show that Plenaris at a dose of 18.8, 9.4, and 4.7 μg ai/seed provided 91, 87, and 84% control of the disease, respectively, relative to untreated seeds. Bion of 25 μg ai /seed and Apron at 50 μg ai/seed provided 62 and 18% control of the disease, respectively. Combining each of the three doses of Plenaris with either Bion of 25 μg ai/seed, Apron of 50 μg ai/seed, or both resulted in enhanced, nearly complete, control of the disease (Fig 9B). All mixtures, except one, exhibited synergistic interactions (SF>1) as shown in Fig 9C.

## Discussion

Infected sunflower seeds and soil-borne oospores serve as the primary inoculum of *P*. *halstedii*. They are responsible for the appearance of only a few systemically infected plants in the field, but the air-borne sporangia dispersed by them drive epidemics forward by infecting other young plants via their apical meristems [5]. Effective control strategy should protect the plants against the disease regardless of the source of inoculum. Because sunflower plants are prone to infection during germination and the early developmental stage of 2–4 true leaves, seed treatment seems to be most appropriate method to provide them with protection against all three types of inoculum. Thus, an effective fungicide(s) applied to the seed should protect the developing seedling from internal infection, from oospore attack of the roots and hypocotyl, and from sporangial attack of the apical meristem of the young seedlings.

The present study reveals that the novel fungicide Plenaris (oxathiapiprolin, OXPT, OSBP-inhibitor) is highly effective against multiple races of *P*. *halstedii* causing sunflower downy mildew. It prevents zoospore discharge from sporangia at a very low dose of 0.0001 ppm ai and prevent cystospore germination of normally released zoospores at this very low dose. When applied as a foliar spray or WSI its MIC values for different isolates range between 0.001–0.1 ppm ai. It is effective not only in preventive but also in post infection applications (1 dpi). These features make Plenaris a suitable candidate for seed treatment against downy mildew in sunflower.

Because oxathiapiprolin is a fungicide attacking the oxysterol binding proteins of the pathogen, and since resistant mutants have already been produced [23, 25, 26], precautions should be taken to avoid the evolvement of resistant mutants. Such precautions include the use of reduced rates of the compound and applying it as a mixture with another fungicide(s) with a different mode of action [17].

In this study, performed in growth chambers, we used both tactics. We mixed Plenaris with Bion and /or MFX and reduced its proportion in the mixtures down to about one tenth of the recommended dose. Seeds were treated with various doses of Plenaris, Bion, Apron, or their 2- or 3- way mixtures. The plants developed from such seeds were inoculated with various isolates of *P*. *halstedii* and the disease was monitored relative to untreated seeds.

It was evident in multiple experiments that reduced rates of Plenaris are less effective than a full rate, but the combination of reduced rates with a full rate of Bion, MFX, or both was as effective as the full rate of Plenaris. This was true in spite of the fact that Bion and/or MFX were poorly effective when applied alone. This suggested that Plenaris synergize with Bion, MFX, or both. The synergistic interaction between the fungicides was calculated by the formula of Wadley or Abbott [35, 36] and was proven positive.

We found similar synergy between oxathiapiprolin and MFX in the control of late blight in tomato [27] and downy mildew in cucumber [28].

Field studies are required to quantitate the efficacy of seed treatment with Plenaris and its mixtures in controlling downy mildew in sunflower. Such studies should be done in various geographical regions where various sunflower hybrids are grown and different races of *P*. *halstedii* prevail.
